# Cellular Senescence, Inflammaging and Cardiovascular Disease

**DOI:** 10.1111/imr.70084

**Published:** 2026-01-16

**Authors:** Lukas Zanders, Denada Arifaj, Julian U. G. Wagner, Stefanie Dimmeler

**Affiliations:** ^1^ Institute of Cardiovascular Regeneration Frankfurt Germany; ^2^ German Center for Cardiovascular Research Partner Site Rhein/Main Frankfurt Germany; ^3^ Clinic for Cardiology University Hospital Frankfurt Frankfurt Germany; ^4^ Cardiopulmonary Institute Giessen Germany

**Keywords:** cardiovascular disease, cellular senescence, heart failure, inflammaging, senolytic, senomorphic

## Abstract

Aging is the most important yet unmodifiable risk factor for cardiovascular disease (CVD). As a result, targeting cardiovascular aging has emerged as a promising strategy to promote long‐term cardiovascular health. This review summarizes current knowledge on the effects of aging within the cardiovascular system as well as systemic processes that modulate them. We highlight the roles of cellular senescence and the senescence‐associated secretory phenotype (SASP), emphasizing their heterogeneous contributions to chronic low‐grade inflammation and tissue remodeling—collectively termed inflammaging. Advances in biomarkers, animal models, and systems biology approaches have deepened our understanding of the interplay between senescence, inflammaging, and cardiovascular dysfunction, including the pivotal role of macrophages in senescent cell clearance. Therapeutic strategies are diverse, ranging from senolytic approaches designed to selectively eliminate senescent cells, to SASP modulation, and interventions targeting chronic inflammation and metabolic dysregulation. Of particular interest, drugs already in clinical use—such as metformin and other anti‐diabetic agents—show beneficial effects on aging‐related pathways, suggesting that their cardiovascular protection may in part reflect anti‐aging properties. Despite these advances, therapies directly targeting senescence and inflammaging to reduce the global burden of CVD remain an urgent unmet need.

## 1 Introduction

Cellular senescence is a complex cellular state, primarily characterized by terminal cell cycle arrest and evasion of apoptosis [1]. When Hayflick and Moorhead first described the phenomenon of replicative senescence in cells of various tissues in 1961, they observed that cardiac fibroblasts were the first cells to undergo senescence [2], potentially indicating an increased susceptibility for cardiac cell populations to become senescent. However, due to a lack of robust biomarkers, 30 years passed until the hypothesis that senescent cells accumulate with aging, was confirmed [3]. More than another decade later, an additional hallmark of senescent cells, the senescence‐associated secretory phenotype (SASP) was first described [4, 5, 6]. The SASP comprises primarily pro‐inflammatory, immunomodulatory and extracellular matrix remodeling components and contributes to the inflammaging phenotype and cardiovascular disease (CVD). With the emergence of biomarkers, animal models and systems biology techniques to systematically investigate complex mechanisms in vivo, our understanding of the interplay between cellular senescence, chronic low‐grade inflammation occurring with aging (inflammaging) and cardiovascular disease has improved. Subsequent studies have shown that these processes are tightly linked and can be influenced by pharmacologic intervention. This review gives an overview of the current knowledge and potential therapeutic applications.

## 2 Chapter I—Cellular Senescence and Cardiovascular Disease

The term cellular senescence describes a cellular state which is characterized by terminal cell cycle arrest. After its first description [2], subsequent work has established various inductors, physiologic functions and detrimental effects, rendering it a complex and heterogeneous condition [7]. Not only the cell‐intrinsic changes, but also the inducing factors, susceptibility and consequences for the microenvironment and the organism vary between cell types and tissues. These findings have led to the understanding that senescence rather represents a continuous spectrum than a categorical state. Importantly, different studies may have investigated distinct senescence states (i.e., “early” or “deep” senescence), which is not reflected in a binominal classification, potentially contributing to heterogeneous or even conflicting results. Cellular senescence contributes to organ aging, which is defined as a progressive decline in function that often precedes age‐associated disease and predicts mortality [8, 9, 10]. While mechanisms such as mitochondrial dysfunction, fibrosis, inflammation, and SASP‐related pathways are shared across organs, organ‐specific aging signatures vary in extent, composition, and dynamics [9, 10, 11, 12]. Transcriptomic and proteomic studies in mice and humans have demonstrated such heterogeneity, with the vasculature emerging as a primary early hotspot of aging in mice [13] and humans [14]. Heart‐specific biomarkers of aging include troponin and NT‐proBNP [9], which however may reflect organ damage, whereas other proteins like GDF15, which are also related to cardiac aging, highlight shared molecular aging signatures across organs and species [12, 15, 16].

### 2.1 Triggers and Functional Consequences of Cellular Senescence

Two broad groups of senescence triggers can be discriminated. First, repeated cell divisions, resulting in replicative senescence, which is mediated by telomere attrition [17], and second, direct stressors, such as radiation, oncogene activation, mitochondrial dysfunction, reactive oxygen species or cytokine stimulation [18]. The initial description of cellular senescence described the replicative exhaustion of cells, in this case human embryonic fibroblasts, after prolonged passaging [2]. This status of terminal cell‐cycle arrest remains the major hallmark of cellular senescence. It is accompanied by the avoidance of undergoing apoptosis through anti‐apoptotic signaling, primarily via the B‐Cell CLL/lmyphoma (BCL‐2) apoptosis regulator family members [19] (refer to chapter 1.5 for more detail). Several senescence inductors accumulate with aging, which are influenced by lifestyle, diseases and their therapies.

Additionally, cellular senescence is an integral mechanism in embryonic development. In contrast to postnatal senescence, which mostly represents a stress response, senescence during development is a programmed process, with similar relevance as proliferation and apoptosis [20]. While showing typical signs of senescence, embryonic senescence encompasses distinct molecular mechanisms and a divergent SASP, primarily encompassing growth factors rather than typical pro‐inflammatory components observed in postnatal senescence [20, 21].

**FIGURE 1:**
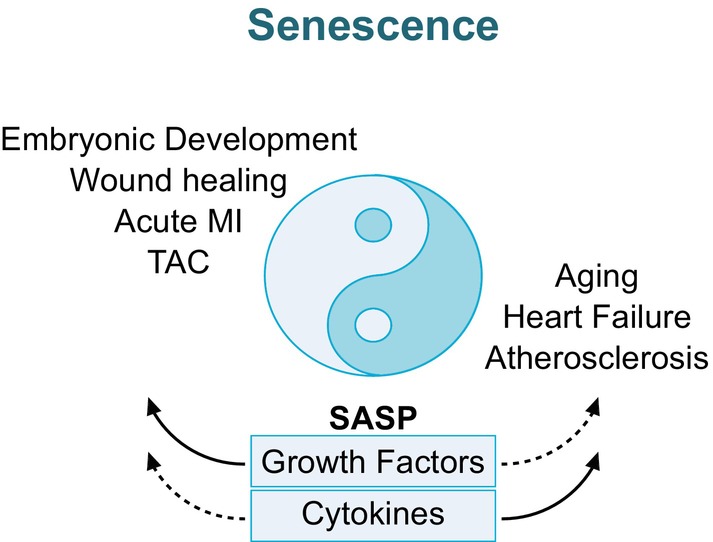
Physiological and pathological effects of senescence.

Despite being mainly investigated as aging and disease‐associated mechanisms, postnatal cellular senescence serves important physiologic functions (Figure 1) with the most prominent role being in tumor suppression. However, this seems to be a double‐edged sword. On the one hand, oncogene activation may induce cellular senescence and the affected cell maintains its senescent phenotype in an autocrine manner via its SASP, especially TGF‐β. On the other hand, the maintenance of this secretome induces tumorigenic effects in a paracrine manner and contributes to the inflammaging phenotype. Additionally, acute (myo‐)fibroblast senescence plays pivotal roles in wound healing and the limitation of organ fibrosis upon acute stressors. Moreover, the SASP of acute postnatal senescent cells can also contribute to tissue regeneration and plasticity to induce a stem cell‐like state [22]. In general, senescence in response to acute stimuli seems to play pivotal roles in tissue regeneration, while chronic accumulation of senescent cells induces detrimental effects (Figure 1).

It remains unclear, if acutely induced senescence affects a distinct cell population which is cleared and eventually followed by the independent induction of chronically induced and accumulating senescent cells, or if cells can transition from the acute (beneficial) to the chronic (harmful) senescent state. In our previous work, we showed that “do no eat me” signals are induced in vascular senescence niches in aged mice, which might induce escape from immunosurveillance and contribute to the chronic accumulation of senescent cells in aging [13]. The investigation of senescence depends on markers to reliably identify senescent cells, which is especially delicate in in vivo models.

### 2.2 Markers of Cellular Senescence

Common features, representing key mechanistic characteristics of cellular senescence are observable in most senescent cells. These include cell cycle arrest, organelle dysfunction‐induced activation of senescence‐associated beta galactosidase (SA‐β‐Gal) [3] and the SASP [4].

#### 2.2.1 Cell Cycle Arrest

Cell cycle arrest is triggered either via the DNA damage response, inducing p53 and p21^Waf1^ (p21) or via direct induction of p16^INK4A^ (p16). While p16 exerts its effects primarily through inhibition of cyclin‐dependent kinase (CDK) 4 and 6, p21 is a broad CDK inhibitor (CDKi). Both mechanisms result in hypophosphorylation of Retinoblastoma (Rb), reducing the transcriptional activity of E2F transcription factors, which is required for cell cycle progression and arrests the cell cycle at G1/S and G2/M (reviewed in [23]).

The widely used markers for cell cycle arrest in senescence p16 and p21 can compensate for each other to induce senescence [24], but show distinct dynamics with differential effects on cell cycle arrest and apoptosis induction. In the time course of senescence, p21 shows early induction, which is followed by p16 expression [25]. p21 dynamics play a pivotal role in cell fate decision and the potential cell cycle re‐entry [26]. As p21 and p53 may also induce apoptosis and quiescence, the commonly used marker for cell cycle arrest in cellular senescence is p16^INK4a^ expression, which can be directly quantified via gene reporters, that is, in the INK‐ATTAC or p16‐3MR mice [8, 27], however p21 reporter mice are also available [28].

#### 2.2.2 Senescence‐Associated Beta Galactosidase (SA‐β‐Gal)

An increased activity at pH 6 of the SA‐β‐Gal is considered the gold standard to detect senescent cells. It is a lysosomal hydrolase, which cleaves β‐d‐galactosides [29]. While being expressed in most cells, higher expression in senescent cells results in detectable activity at the suboptimal pH 6, which is distinct from the typical optimum at pH 4 in non‐senescent cells [3]. Its up‐regulation is part of both increased lysosomal biogenesis and lysosomal accumulation of the enzyme and SA‐β‐Gal up‐regulation can be caused by the CDKi cascade [29]. However, increased activity is not specific to senescent cells, as many tumors show similar activity levels at pH 6 [30, 31]. Additionally, macrophages, which have a high lysosomal β‐galactosidase activity per se, can show positive activity at pH 6 while not showing other signs of senescence [32].

#### 2.2.3 Senescence‐Associated Secretory Phenotype (SASP)

Senescent cells significantly change their secretome. It encompasses pro‐inflammatory, pro‐fibrotic and growth‐inducing proteins, nucleic acids, lipids and extracellular vesicles and thereby alters the senescence niche and exerts systemic effects (the broader composition is reviewed in [33]). The SASP was first described in 2008 by three independent groups as a primarily pro‐inflammatory and protein‐based secretome and our understanding of the composition and effects has vastly improved over the past years [4, 5, 6, 33]. The SASP plays pivotal roles in local anti‐tumor effects and autocrine and paracrine induction and maintenance of the senescence state [34, 35]. The best characterized part of the SASP is proteins involved in pro‐inflammatory signaling, especially chemokines and cytokines, as well as extracellular matrix remodeling proteins, that is, matrix metalloproteinases. The pro‐inflammatory components of the SASP are mainly induced via the transcription factors nuclear factor of kappa light polypeptide gene enhancer in B‐cells (NF‐kB) and CCAAT enhancer binding protein beta (C/EBPb), inducing the expression of cytokines such as interleukins (IL) 6 and 8 in senescent cells. This effect has been reported to be primarily regulated by IL‐1α [36], which is particularly important in oncogene‐induced senescence [37]. While these effectors maintain the senescence state and hence contribute to tumor suppression of the senescent cell [6], they also contribute to systemic pro‐inflammatory effects and hence systemic tumorigenic effects [37]. C‐X‐C motif ligands (CXCL) are chemokine components of the SASP, which mediate immune cell infiltration and activation, induce cancer cell proliferation and contribute to senescence maintenance via binding to their respective receptors, with a particularly important role for CXCR2 [5, 38, 39].

In addition to pro‐inflammatory components, various growth factors are contained in the SASP. Within this group, Transforming Growth Factor beta (TGF‐β) and Growth Differentiation Factor 15 (GDF‐15) play pivotal roles. GDF‐15 is a core SASP component which associates with aging, frailty and age‐related diseases [15, 40, 41, 42]. It belongs to the TGF‐β cytokine superfamily and plays roles in embryonic development, tissue repair but also cancer progression and metastasis [43, 44]. Moreover, it has been associated with poor outcomes in various cardiovascular diseases, such as coronary artery disease [45], atrial fibrillation [46] and heart failure with reduced and preserved ejection fraction [47]. TGF‐β is a prototypical pro‐fibrotic growth factor, which induces fibroblast proliferation and activation [48] and is required for endothelial to mesenchymal transition and activation in the heart [49]. TGF‐β plays a central role in the SASP‐mediated induction of senescence in embryogenic senescence, as well as postnatal senescence in the liver and the tumor microenvironment [50, 51].

Furthermore, the SASP changes with different senescence states, with higher expression levels of typical components, such as *IL1A*, *CCL2* and *TNFRSF11B* observed in deep senescence states than in early states. More recent data showed that late senescence induces the retrotransposable element LINE1, inducing type 1 interferon responses, which are required for the maintenance of senescence, suggesting a time‐dependent program of SASP composition [52].

Interestingly, high SASP expression can contribute to an immune‐suppressive signature, with increased T regulatory cells, macrophages with immune‐suppressive properties and exhausted T cells which is dependent on TGF‐β [50]. Additionally, senescent niches express high levels of immune inhibitory signals, potentially facilitating this phenotype [13]. This might indicate that insufficient clearance and accumulation of senescent cells in chronic senescence is a consequence of TGF‐β and SASP‐mediated effects.

#### 2.2.4 Combinatorial Approaches and Senescence Scores

The primary biomarkers p16 and SA‐β‐Gal have allowed for identification and a thorough mechanistic understanding of senescence in vitro. Given the relevant heterogeneity in context and niche‐dependent functions, phenotypes and paracrine effects of senescence in vivo, “omics” approaches, especially single‐cell and single‐nucleus sequencing, seem promising modalities to investigate senescence in vivo. However, the identification of senescent cells is hampered by the usually low expression of *CDKN1A/Cdkn1a* (encoding for p21) and *CDKN2A/Cdkn2a* (encoding for p16), which are hence prone to drop‐out effects. Therefore, gene sets, databanks and scoring approaches are required to characterize cellular senescence in development, aging and disease.

The SeneQuest [53], CellAge [54] and SenMayo [55] databases, which are curated sets of senescence‐associated genes, may facilitate such approaches. However, as these datasets lack cell type specificity, they seem limited in in vivo settings. There is an urgent need for organ and cell type‐specific characterization of cellular senescence to facilitate therapeutically targeting the detrimental effects of cellular senescence in general and in the cardiovascular system specifically.

### 2.3 Cellular Senescence in Cardiovascular Disease

Cardiovascular disease and cellular senescence within the myocardium and vasculature share a bidirectional relationship. The age‐associated accumulation of senescent cells predisposes individuals to cardiovascular disease and adverse remodeling, but cardiovascular diseases are associated with increased senescence, potentially contributing to aging signatures.

Senescent cells accumulate in the aging heart, primarily within the vascular niche and associate with the myocardial aging hallmarks fibrosis and diastolic dysfunction [13, 56, 57]. Interestingly, the murine senescence models bearing progeria‐driver mutations or telomerase deficiency typically die from heart failure [58, 59] and patients with Hutchinson‐Gilford Progeria Syndrome develop early and severe atherosclerotic disease, which is the primary cause of death in these patients [60]. Targeting p16 to induce senolysis in mice, however, does not rescue senescence in the heart, pointing towards a p16‐independent pathomechanism in these models [8]. This section gives an overview of the role of senescence in the most frequent age‐associated cardiovascular diseases: atherosclerosis and heart failure.

#### 2.3.1 Cellular Senescence in Atherosclerosis

Apart from genetic predisposition, such as familial hypercholesterinemia or hyperlipoproteinemia (a), atherosclerosis is a typical age‐associated disease. Aged a therosclerosis‐prone *Ldlr* ^−/−^ mice show a myeloid skewing in the blood with an overall decreased abundance of lymphoid cells. On the other hand, within atherosclerotic plaques, aging associates with an increase in CD4+, CD8+ and CD4 + CD8+ T‐cells. Aging induced a shift in the macrophage population with an increase in Il1b^hi^ macrophages and a higher expression of MMPs [61] (see also Chapter 2 for further details on macrophage‐related aging phenotypes).

Atherogenesis involves senescence of various cell types, including endothelial cells (EC), vascular smooth muscle cells (VSMC), macrophages and foam cells. Endothelial cell senescence contributes to risk factors associated with atherosclerosis, especially insulin resistance [62] and endothelial dysfunction [63], which are reversible through senolysis [64]. Senescent endothelial cells act as a feed‐forward mediator of senescence via SASP components, including IL‐1α, IL‐6, IL‐8 and CCL2, but also proteins associated with matrix remodeling and growth factor signaling, hence forming a pro‐inflammatory and pro‐atherogenic milieu [62]. Mature atherosclerotic plaques show a high burden of senescent endothelial cells in humans [65] and a model of endothelial cell senescence in an *Apoe* ^−/−^ background shows accelerated plaque progression and inflammatory responsiveness via enhanced VCAM1 promoter accessibility for NF‐B [66]. Importantly, senescent ECs show increased monocyte adhesion [67] which in combination with increased endothelial permeability [68] may facilitate tissue infiltration with bone marrow derived macrophages.

Senescent foamy macrophages, as indicated by p16‐induction, are enriched in atherosclerotic plaque early in the course of atherogenesis in *Ldlr* ^−/−^ mice and genetic senescence clearance attenuates plaque progression. Senescent cells are major contributors to the pro‐inflammatory plaque milieu and plaque remodeling via the expression of matrix metalloproteinases [69]. As part of the vascular niche, VSMC undergo age‐associated senescence [13]. Senescent VSMC exert a typical pro‐inflammatory SASP profile, including IL1α, IL6, IL8 and CCL2 [25]. A comprehensive review on cellular senescence in atherosclerosis is provided in [70].

#### 2.3.2 Cellular Senescence in Heart Failure

The most frequent forms of heart failure, heart failure with preserved ejection fraction (HFpEF), as well as ischemic cardiomyopathy, the most common etiology of heart failure with reduced ejection fraction (HFrEF), occur strongly age‐associated. Early work on senescence in human heart failure showed increased senescence in presumed (c‐kit positive) cardiac stem cells [71, 72]. However, with the increasing evidence against the existence of relevant cell populations with stem cell properties in the adult human heart, it is likely, that the increased abundance of senescent c‐kit positive cells in the failing heart represent EC [73]. This is in line with the finding that senescence‐prone mice on western diet develop endothelial senescence and heart failure with preserved ejection fraction [74].

In human idiopathic dilated cardiomyopathy, senescence spatially overlaps with perivascular fibrosis. Similarly, in mouse models of fibrotic heart disease, fibrosis, a key feature of chronic myocardial disorders, was also associated with cellular senescence, suggesting that fibroblast senescence may contribute to the progression of heart failure. However, the role of fibroblast senescence in myocardial fibrosis remains controversial. On the one hand, cellular senescence correlates with myofibroblast‐driven perivascular fibrosis in the Angiotensin‐II hypertension model and left ventricular pressure overload via transaortic constriction (TAC) [75]. This observation is, however, not informative on the consequences of fibroblast senescence, which are controversial. Using genetic depletion of senescence via deletion of p53 and p16 in TAC‐treated mice results in increased perivascular fibrosis [75]. Treatment with the senolytic agent navitoclax 4 weeks after initiation of AngII treatment in WT mice, however, reduces fibrosis and improves diastolic dysfunction [76]. The relevant difference between these models may lie in the timing of senolysis. While global deletion of p53 and p16 prevents the development of cellular senescence from the beginning of LV pressure overload, treatment with navitoclax 4 weeks after induction of AngII treatment clears senescence in the later course of fibrosis. This may indicate anti‐fibrotic effects of acute myofibroblast senescence, while chronic fibroblast senescence may be pro‐fibrotic, which is in line with findings from wound‐healing studies [27].

Increased fibroblast‐derived TGF‐β induces cardiomyocyte senescence and dysfunction in LV‐pressure overload [77]. Cardiomyocyte senescence induced by acute myocardial infarction is associated with impaired regeneration and angiogenesis [78, 79], which is in accordance with the atypical SASP of senescent cardiomyocytes which is mainly characterized by growth factors, which induce paracrine senescence in other myocardial cell populations [80].

As it is a well‐defined characteristic of age‐associated cardiovascular disease, targeting cellular senescence represents a promising therapeutic target.

### 2.4 Therapeutic Approaches Targeting Senescence

#### 2.4.1 Experimental Approaches With Senolytics

The survival and accumulation of senescent cells rely on pathways to evade apoptosis. These pathways are targeted by so‐called senolytic agents or senolytics. By inhibiting the anti‐apoptotic pathways, senolytics induce apoptosis in senescent cells and hence induce senescence clearance [81]. The best characterized mechanism for apoptosis evasion is via anti‐apoptotic BCL‐2 family member signaling. This complex regulatory network contains pro‐ and anti‐apoptotic mediators, regulating intrinsic apoptosis via mitochondrial outer membrane permeabilization (reviewed in [82]). Upstream regulators of the BCL‐2 family, which are activated in senescent cells and exert anti‐apoptotic effects are ephrins, Src kinases, PI3KCD, plasminogen activator inhibitors (i.e., PAI‐1/Serpine1).

Additionally, senescent cells can evade extrinsic apoptosis, that is, viaPAI, which inhibits death receptors and caspase 3 [83]. Importantly, there is relevant heterogeneity between the induced pathways and not all senescent cells induce similarmechanisms, which also results in heterogeneous responses to different senolytic agents [81].

Currently available senolytics are repurposed compounds, with no available drugs being specifically developed or approved for their usage as senolytics. The best characterized senolytic agents are the tyrosine kinase inhibitor dasatinib, which is clinically used for treating chronic myeloid and acute lymphoblastic leukemia [84, 85]. The senolytic effect stems from its inhibition of Src family kinases and ephrin‐induced anti‐apoptotic effects [86, 87]. While showing good senolytic capacity in preadipocytes, given as a single drug it does not induce apoptosis in senescent endothelial cells. Therefore, it is commonly combined with the plant flavonoid quercetin, which shows a complementary profile (dasatinib + quercetin; D + Q), especially including additional targeting of senescent endothelial cells [56, 88]. In addition to quercetin, additional natural products and phytochemicals have shown the ability to act as senolytics and interfere with SASPs such as fisetin, curcumin, and procyanidin C1 [89, 90, 91]. Fisetin inhibits the PI3K/Akt/mTOR pathway and shows some specificity for senescent endothelial cells [89]. The compound navitoclax exerts effects on various cell types and is a direct inhibitor of the anti‐apoptotic Bcl‐2 family members Bcl‐2, Bcl‐xL and Bcl‐w [92].

Senolytics have demonstrated that selectively clearing cardiac senescent cells can mitigate the detrimental consequences of cardiac aging and related diseases [93]. We and others have shown that senolytic treatment in aged mice rescues aging‐induced adverse remodeling in the heart using navitoclax [80], D + Q [56, 57, 88] or fisetin [13]. Navitoclax treatment improves left ventricular remodeling and post‐myocardial infarction survival [94, 95]. D + Q has shown promising results to reduce atrial fibrillation inducibility after myocardial infarction and arrhythmia rates with aging [56, 96].

In the murine Angiotensin II infusion model, treatment with navitoclax attenuates the pro‐fibrotic response through clearance of senescent fibroblasts [76] (see also chapter 1.3.2). Interestingly, D + Q has been found to prevent mitochondrial DNA–induced inflammation and prolong the survival of cardiac allografts [97].

The effects of senolysis in late atherosclerosis remain controversial. One experimental study reported reduced lesion size in *ApoE* ^−/−^ mice upon navitoclax treatment [98], while another demonstrated increased lesion size and induced mortality [99]. The latter trial used very high navitoclax doses, which might have induced toxicity. In *Ldlr* ^−/−^ mice, the same dosage given for a significantly shorter period reduced atherosclerosis progression [69]. All three studies demonstrate smooth muscle cells and macrophages/foam cells to be the main senescent populations in atherosclerosis.

The pioneering development of chimeric antigen receptors (CAR‐)T cells has expanded opportunities for cell‐specific targeting in cardiovascular medicine. Beyond preclinical studies showing that engineered CAR‐T cells or CAR‐macrophages directed against activated fibroblasts can improve cardiac function in injury models [100, 101], similar strategies may be envisioned to selectively eliminate senescent cells. Indeed, natural killer group 2D (NKG2D‐) CAR T cells have been shown to eliminate senescent cells in aged mice and nonhuman primates [102], while other studies demonstrated prophylactic and durable efficacy of senolytic CAR‐T cells against age‐related metabolic dysfunction [103]. Designing targeted approaches to eliminate senescent cells within the cardiovascular system could thus provide a foundation for innovative and highly specific therapeutic strategies. Given the complexity and high treatment costs associated with these therapies, a prerequisite for clinical use will be the identification of suitable patients, who are at high risk for adverse outcomes and will very likely profit from targeted senolysis.

Table 1 gives an overview of the currently available work on the experimental use of senolytics in cardiovascular disease.

**TABLE 1:** Representative preclinical studies of senolytic therapies in cardiovascular disease models.

Study (author, year)	Organ/model	Senolytic	Dose schedule	Duration	Outcome/effects	Toxicity
Mehdizadeh et al. (2024) [95]	Heart‐Sprague Dawley rats 2 months, Myocardial Infarction (MI)	D + Q	2*3× with a 2‐week interval in between, 1 week recovery	4 weeks	Reduced AF inducibility in MI rats and attenuated LA fibrosis by clearing senescent fibroblasts and endothelial cells.	
Dookun et al. (2020) [94]	Heart, 3 months mice, ischemia–reperfusion injury	Navitoclax (50 mg/kg/day)	Post ligation/reperfusion, 7 consecutive days, 4 weeks recovery	7 weeks	Improved myocardial remodeling, left ventricular function and reduced post MI‐mortality	Reversible reduction of platelet count after the stop of treatment
Walaszczyk et al. (2019) [93]	Heart, 18–20 months mice, Acute Myocardial Infarction (AMI)	Navitoclax (50 mg/kg/day)	7 consecutive days	1 week	Improved the maintenance of cardiac function following MI	No overt toxicity
Anderson et al. (2019) [80]	Heart‐ 23 months mice	Navitoclax	7 consecutive days every other week	3 weeks	Reduced ventricular fibrosis and hypertrophy.	
McDougall et al. (2019) [57]	Heart‐ 24–32 months mice	D + Q	3× every other week	8 weeks	Attenuates impaired heart regeneration	No overt toxicity
Zhu et al. (2015) [88]	Heart‐ 24 months mice	D + Q	1×, 5 days recovery	6 days	Improved LV EF, vascular endothelial function	No overt toxicity
Iske et al. (2020) [96]	Heart‐ aged donor mice (18–22 months)‐cardiac transplantation into young recipients	D + Q	3× every week	1 month	Reduced pro‐inflammatory T cell response and prolonged survival of cardiac allografts from aged donors	No overt toxicity
Wagner et al. (2023) [56]	Heart‐ 18 months mice	D + Q (5 mg/kg/day; 50 mg/kg/day)	3× every other week	8 weeks	Reduced endothelial senescence, restored ventricular innervation, stabilization of heart rate variability and reduction of arrythmic tendencies.	No overt toxicity
Rodriguez‐Morales et al. (2025) [13]	Heart‐ 18 months	Fisetin (100 mg/kg/day)	3× every other week	8 weeks	Reduced ventricular fibrosis, improved diastolic function.	No overt toxicity
Jia et al. (2020) [76]	Heart‐ AngII infusion	Navitoclax			Improved LVEF, conduction velocity, reduced fibrosis, arrhythmia succeptibility	No overt toxicity
Garrido et al. (2021) [97]	Athero‐ *ApoE* ^−/−^ HFD 15 weeks	Navitoclax (50 mg/kg/day)	5 consecutive days, 3 weeks recovery, 3 cycles	12 weeks	Reduced atherosclerosis lesion area, absolute core size	Thrombocytopenia, lymphopenia
Karnewar et al. (2024) [98]	Athero‐ *ApoE* ^−/−^ HFD 18 weeks	Navitoclax (100 mg/kg/day)	5 consecutive days, 2 weeks recovery, 3 cycles	9 weeks	Increased atherosclerotic lesion area, increased mortality	Increased mortality
Childs et al. (2016) [69]	Athero‐ *Ldlr* ^−/−^ HFD 9 days	Navitoclax (100 mg/kg/day)	9 consecutive days	9 days	Decreased lesion area	No report on toxicity

#### 2.4.2 Studies in Humans

Although preclinical studies indicate that senolytics can improve cardiac function in aged animals, evidence in humans remains limited, and no therapies have yet been shown to conclusively slow or reverse age‐related decline in heart function. Nevertheless, several early‐phase clinical trials are now investigating fisetin in older adults, targeting vascular aging and arterial diseases (Table 2). For example, one (NCT06399809) is assessing whether fisetin can reduce senescent cell burden and improve functional outcomes in individuals with peripheral artery disease. Additional trials are evaluating intermittent fisetin treatment to enhance vascular endothelial function and reduce aortic stiffness, as well as its safety, pharmacokinetics, and tolerability in healthy and older adults with multiple chronic conditions (NCT06133634, NCT06431932). Most evidence on the effects of senolytics comes from studies in age‐related neurodegenerative diseases, as reviewed in [106]. As these results may also be instrumental for developing cardiovascular‐targeted strategies, and with the brain–heart interaction gaining increasing recognition, these findings are additionally summarized in Table 2. While most studies are ongoing, phase I clinical trials investigating D + Q showed feasibility and trends towards improved surrogate markers in patients at risk or with mild Alzheimer's disease [107, 108].

**TABLE 2:** Representative clinical studies with senolytics.

Trial ID/acronym	Condition/population	Senolytic agent(s)	Design	Dose/regimen	Primary outcomes/endpoints	Status/notes
NCT05422885 (STAMINA study)	Old adults at risk of Alzheimer Disease	D + Q	Preventive, Single‐arm, open‐label, pre–post pilot study	D (100 mg/day) + Q (1250 mg) for 2 consecutive days, every 2 weeks six cycles up to 12 weeks	Feasible and trends in cognitive assessment (subgroup) and biomarkers	Completed and published [104]
NCT04063124 (SToMP‐AD study, pilot study)	Mild Alzheimer's disease	D + Q	Phase I, open labeled, pilot trial	D (100 mg) + Q (1000 mg); daily for two consecutive days followed by a 14‐day no‐drug period, six cycles up to 12 weeks.	Feasible and trends in biomarkers	Completed and published [105]
NCT05838560	Schizophrenia and treatment‐resistant depression	D + Q	Phase II, open‐label, pilot trial	D (100 mg/day) + Q (1250 mg/day) for 2 consecutive days weekly	Safety & Feasibility, senescence markers, MRI	Primary Completion estimated 01/01/2026
NCT04785300 (ALSENLITE study)	Mild Cognitive Impairment or Alzheimer's disease	D + Q	Phase II, open‐label, pilot	D (100 mg/day) + Q (1250 mg/day) for 2 consecutive every 2 weeks for 6 cycles	Safety & tolerability	Recruiting
NCT04685590 (SToMOP‐AD study)	Early Stages Alzheimer's disease	D + Q	Phase II, multi‐ site, randomized, double‐blind, placebo controlled trial	D (100 mg/day) + Q (1000 mg/day) for 2 consecutive days every 2 weeks for 6 cycles	Feasibility and safety	Active, not recruiting
NCT04907253	Coronary Bypass Surgery	Quercetin	Phase II, double‐blind, placebo‐controlled randomized study	500 mg/day starting 2 days before surgery and up to 10 days after the surgery	Health Status, Surgery‐associated inflammatory and senescence markers	Active, not recruiting
NCT06399809 (FIRST)	Peripheral Artery Disease	Fisetin	Phase I, pilot Placebo controlled, randomized trial	20 mg/kg/day for 2 consecutive days every 2 weeks in total of 4 months	Improvement in 6 min walk distance	Recruiting
NCT06133634	Arterial Stiffness in Aging	Fisetin	Phase II, randomized, double‐blind, placebo controlled	2 mg/kg/day for 3 consecutive days, every 2 weeks for 1 month	Endothelial function, pulse wave velocity	Recruiting

While these senolytic interventions show encouraging neuro and cardioprotective effects, they are not without potential side effects, including cytopenias, gastrointestinal symptoms, and off‐target toxicity. Recently, galactose‐modified senolytic prodrugs have been developed, which take advantage of SA‐β‐Gal activity in senescent cells hence increasing specificity. Examples are JHB75B, a duocarmycin derivate, which belongs to a group of highly cytotoxic antibiotics [109], and Nav‐Gal, a navitoclax prodrug, which carries a reduced risk of inducing cytopenias [110]. Overall, the currently available data call for randomized controlled trials with clinically relevant endpoints to investigate beneficial effects of senolysis in at‐risk aging populations and patients with cardiovascular disease.

#### 2.4.3 Senomorphics

**FIGURE 2:**
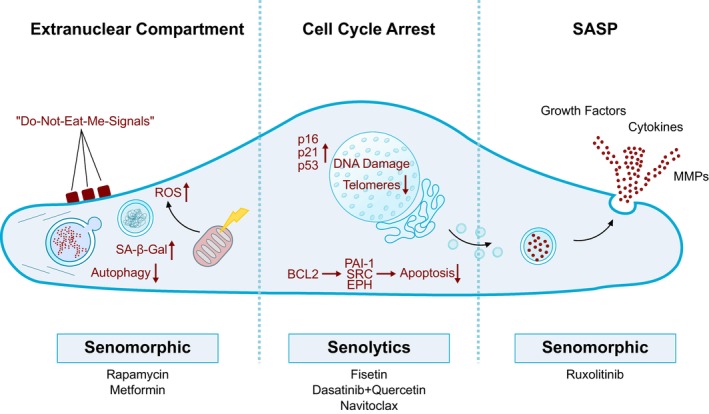
Mechanisms and targets of cellular senescence.

Senomorphics are a class of pharmacological agents that target the detrimental effects of senescent cells without inducing apoptosis, distinguishing them from senolytics. These compounds primarily act by modulation of the SASP. They target signaling pathways involved in SASP regulation, including mTOR, AMPK, NF‐κB, p38 MAPK, and JAK/STAT (Figure 2). Rapamycin and metformin are among the most extensively studied senomorphics. Rapamycin suppresses mTOR signaling, thereby reducing oxidative stress and enhancing autophagy [111]. In preclinical studies, it has been shown to delay the age‐related decline in both systolic and diastolic cardiac function in mice [112] and dogs [113]. Improvements in cardiac performance have also been reported in a mouse model of progeria [111]. Moreover, treatment with rapamycin further alleviated oxidative stress and vascular dysfunction in aged mice [114].

Beyond its widespread use in diabetes management, metformin is now being explored for its ability to slow biological aging. The ongoing TAME trial investigates if it can reduce the burden of age‐related diseases [115]. Clinical trials suggest benefits beyond glucose control, as older individuals with impaired glucose tolerance showed improvements in both muscle and fat tissue function when treated with metformin [116]. Mechanistic work in experimental models links these effects to enhanced autophagy in vascular cells, activation of AMPK, and suppression of stress responses such as mTOR signaling and endoplasmic reticulum stress in the heart [111]. Remarkably, long‐term administration in primates slowed aging across diverse organs, such as the cardiovascular system, the brain and skin, by mitigating inflammation, senescence, and epigenetic alterations [117]. While these data are promising, clinical confirmation is still required to establish metformin as a therapy that truly modifies human aging.

Ruxolitinib, a JAK1/2 inhibitor, is another well‐studied senomorphic. By blocking JAK/STAT signaling, it reduces the secretion of pro‐inflammatory SASP factors and alleviates systemic inflammation, thereby improving tissue function in preclinical models of aging [118]. Other compounds, including p38 MAPK inhibitors (e.g., SB203580), glucocorticoids like dexamethasone, and sirtuin activators such as resveratrol, have also been shown to attenuate SASP production and restore cellular function.

Taken together, the currently available data suggest that cellular senescence is a hallmark of both cardiovascular aging and disease, which can be therapeutically targeted. As the cell types and mechanisms involved vary between diseases, future work has to identify suitable compounds to target the distinct deleterious populations and effects to guide new precision‐medicine approaches in cardiovascular aging and disease.

## 3 Chapter 2—Inflammaging

Inflammaging refers to the critical interplay of inflammatory processes during aging. It is characterized by a long‐lasting, chronic, low‐grade activation of the inflammatory response that occurs in the absence of pathogens or external triggers. Paradoxically, age‐associated low‐grade immune activation is accompanied by a reduction in effective and specific responses to antigen stimulation or viral defense, termed immunosenescence. Inflammaging is observed across species, including great apes, but may be influenced by lifestyle factors [119]. This age‐associated dysregulation of the immune system contributes to the functional decline of organs and increases susceptibility and progression of diseases with aging. Various organs, including the heart, vasculature, brain, adipose tissue, lung, liver, and kidney, shift towards a proinflammatory state during normal aging, posing a heightened risk for non‐communicable diseases [11, 12]. However, it is important to note that inflammaging is not necessarily exclusively pathological; it may represent a delicate balance between an adaptive response and a harmful, excessive reaction, potentially reflecting the body's attempt to make the best of an unfavorable physiological situation.

### 3.1 Inducers and Immune Dysregulation in Inflammaging

**FIGURE 3:**
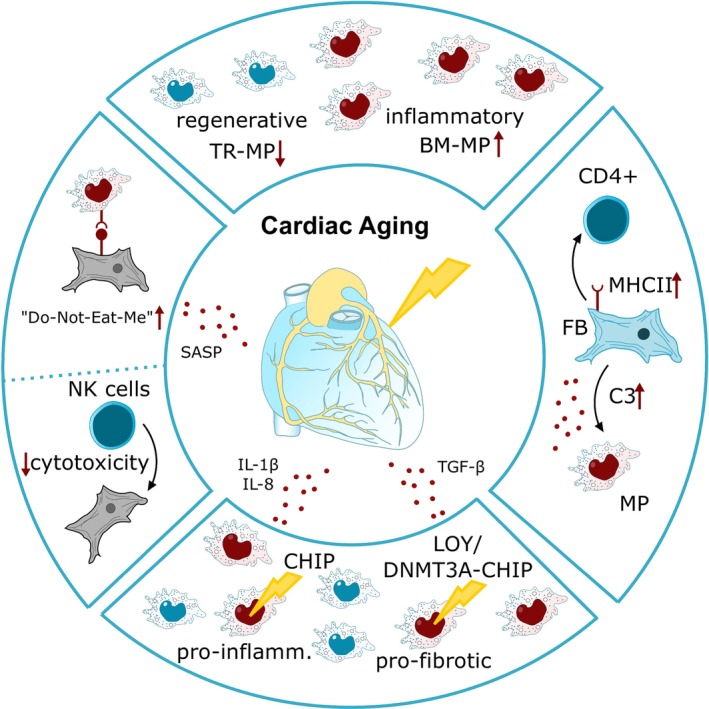
Contributors to cardiac inflammaging.

Inflammaging is driven by multiple factors that contribute to the progressive deterioration of the immune system [120] (Figure 3). Major contributors include the accumulation of cellular debris and damaged cells that are not efficiently cleared. This is particularly relevant for senescent cells, which accumulate in tissues and continuously release a pro‐inflammatory SASP. Additionally, impaired organelle function, such as mitochondrial dysfunction or reduced autophagy, promotes the persistence of activated, dysfunctional cells and the release of damage‐associated molecular patterns (DAMPs) [7]. Systemic changes, including alterations in gut microbiota composition and increased intestinal permeability, can further enhance age‐associated activation of the innate immune system [121]. Alterations in the immune system during aging occur in different cell populations. Aging induces a myeloid skewing, characterized by increased numbers of monocytes and neutrophils, while lymphocyte populations are reduced [61, 122].

Myeloid cells in aged individuals display excessive cytokine production but paradoxically have diminished functional capacity [123]. Specifically, monocytes and macrophages lose their phagocytic ability to clear senescent cells and tissue debris, leading to unresolved, chronic inflammation. Mechanistically, impaired autophagy plays a critical role in the acquisition of major aging features in macrophages [124] and the finding of shortened telomeres might indicate senescence in monocytes [123]. Additionally, the monocyte composition changes with aging, with a skewing towards intermediate and non‐classical monocytes, which exert increased pro‐inflammatory responses upon TLR4 stimulation [123].

Among the molecular regulators of age‐associated changes within the immune system is the NLRP3 inflammasome and the prominent role of IL‐1β and IL‐8 in the SASP also indicates a central role in senescence‐induced inflammaging. NLRP3 contributes to the accumulation of aged adipose B cells in lymphoid clusters in the adipose tissue, contributing to metabolic dysfunction [125], insulin resistance, and cardiovascular disease in aging [126] and is a key mediator of the pro‐inflammatory effects of age‐associated clonal hematopoiesis (see chapter 2.4). A wide range of age‐associated diseases, ranging from cognitive decline [127] and Alzheimer's disease [128] to hypertrophic heart disease [129] are attenuated by NLRP3 inhibition.

In the context of defunct debris clearance and accumulation of aberrant and senescent cells, there seems to be a change in immunosurveillance with aging. Senescent cells have been reported to evade NK cell‐mediated clearance [130]. Although major lymphoid lineages decline with increased age, the age‐associated change in NK subtypes remains controversial (reviewed in [131]). Previous studies demonstrated increased CD56^dim^ and decreased CD56^bright^ populations with aging in blood, which would indicate an overall functional shift from cytotoxic towards a pro‐inflammatory phenotype [132, 133]. However, a more recent comprehensive work has not found any age‐associated changes in NK subpopulations [134]. NK cells rely on cytokine production and cytotoxic activity to fulfill their role in immunosurveillance. Interestingly, NK cells do not lose their capacity for cytokine production but rather lose the stimulus with decreasing circulating IL‐2 plasma concentrations with age [135]. The cytotoxic capacity of NK cells decreases with age [133], which associates with increased risk for cancer [136].

In addition, adaptive immune cell types, such as T cells, B cells, and dendritic cells, are altered and functionally compromised with age (reviewed in [137]). Particularly, aging is associated with profound changes in T cell subsets with low CD4 and high CD8 cells [138, 139, 140]. Within the CD4^+^ T cell population, the frequency of naïve cells is reduced in the elderly, whereas effector memory and terminally differentiated CD4^+^ T cells are increased compared with adults [139]. In the CD8^+^ T cell compartment, a large‐scale transcriptomic study spanning nine decades revealed dynamic shifts in both population composition and gene expression [141]. T cells exhibit altered expression of genes involved in T cell receptor proliferation, signaling, cytokine/chemokine networks, cytotoxicity, and metabolic regulation [138]. Particularly, effector memory T cells in aged individuals often display exhaustion and senescence features. They lose the capacity to migrate to lymphoid organs such as lymph nodes and the spleen, instead acquiring the ability to infiltrate non‐immune tissues. T cell senescence and exhaustion seem to co‐occur with aging [142] and regulatory T cells can induce responder T cell senescence. Senescent T cells secrete the SASP components IL‐6 and TNFα [143], while typical cytokines secreted by activated T cells remain unchanged [144]. This shift in T cell distribution and function compromises immunosurveillance, promotes autoimmune responses, and impairs the repair of biological barriers—together fostering inflammaging.

### 3.2 Inflammaging and the Aging Heart

Inflammaging also affects the heart, where aging alters immune cell composition and function, leading to changes in both steady‐state and inflammatory responses. Particularly, the composition, phenotype and function of cardiac macrophages both at steady state and during inflammation are altered by age [145]. While mice aged 12–15 months show only limited changes in myeloid cells [146], older mice (18–24 months), corresponding to humans over 55 years, display significantly higher numbers of total leukocytes (CD45^+^) and CD11b^+^ myeloid cells, with specific increases in neutrophils and both Ly6C^high^ and Ly6C^low^ monocyte subsets in the heart compared to young mice [145]. Consistently, we reported an increase in bone marrow–derived macrophages in aged mouse hearts, accompanied by a decline in tissue‐resident, reparative CD68^+^Lyve1^+^ macrophages [13]. Recent studies further suggest that aging affects not only cell numbers but also macrophage function: *proto‐oncogene tyrosine‐protein kinase MER* (*MERTK*) mRNA expression is reduced in cardiac‐resident macrophages from old mice [145], while the expression of “do not eat me” proteins such as SIRPA is increased [13], consistent with a decline in efferocytic capacity and clearance function. This dysregulation has also been observed in spatial transcriptomic analyses of human hearts demonstrating accumulation of this SIRPA+ macrophages population together with senescent cells in the perivascular area [13]. In addition, myeloid‐derived suppressor cells (MDSCs), a population of immature myeloid cells with suppressive function on adaptive immune responses, are affected by aging. Although the total number of monocytic MDSCs in the hearts of old mice at steady state does not differ from that in young mice, MDSCs from aged animals exhibit impaired ability to suppress T‐cell proliferation [145], a defect that may contribute to chronic inflammation.

Aging‐associated myocardial impairment additionally coincides with reduced CD4 T cells, shifts in the subtypes of tissue‐resident leukocytes and the accumulation of activated CD4^+^ Foxp3^−^ IFN‐γ^+^ T cells in heart‐draining lymph nodes [146]. The increased numbers may be the consequence of increased homing, as adoptive cell transfer of T cells from aged animals induced increased cardiotropism of T cells obtained from old donors [146].

Finally, aging not only changes the basal phenotype but also the dysfunctional activation of immune cells, which has been associated with an impaired prognosis after MI in the elderly [147, 148].

#### 3.2.1 Consequences of Immune Cell Dysregulation in the Aging Heart

Overall, age‐related changes in innate immune function can contribute to heart failure through multiple mechanisms, including altered responses to infections [149] or direct effects of aging on the immune system.

Cardiac macrophages play a key role by regulating fibrosis and thereby promoting diastolic dysfunction—a hallmark of the aging heart [150]. With age, there is a shift towards pro‐inflammatory bone marrow–derived macrophages and a decline in reparative cardiac resident macrophages, altering the cardiac microenvironment and driving fibroblast activation, which in turn increases collagen deposition. This process may be further exacerbated by cellular senescence, as senescent macrophages release TGF‐β, thereby enhancing fibroblast activation [151]. Pioneering studies by Hulsman et al. demonstrated a crucial role of macrophages in facilitating electrical stability in the heart [152]. The reduced number of cardiac resident macrophages, which mediate this effect, in aging may contribute to impaired atrioventricular conduction often observed in the elderly. In line, others reported that aging results in chronotropic incompetence and disturbed repolarization after lipopolysaccharide injection [145].

Finally, one should not forget that macrophages are also important players in the resolution of fibrosis. Recent studies have highlighted their role in reverse remodeling of fibrotic tissue [153, 154]. Thereby, macrophages contribute to the resolution and clearance of fibrotic material following the relief of cardiac stress, such as unloading after pressure overload. Since the reversal of fibrosis depends on the clearance capacity of macrophages, a function that is impaired during aging [155], it is plausible that the accumulation of aged macrophages may hinder this reparative process as discussed. Cardiac macrophages promote diastolic dysfunction via IL‐10‐mediated fibroblast activation in aged mice, an interesting finding, as IL‐10 is anti‐inflammatory, but a typical SASP component in macrophage senescence [150, 156].

Fibroblasts themselves may fuel inflammaging. In the aging mouse heart, mesenchymal fibroblasts adopt a pro‐inflammatory phenotype, producing elevated levels of chemokines, primarily monocyte chemoattractant protein‐1 (MCP‐1), which promotes leukocyte recruitment into the heart [157]. In addition, aged cardiac fibroblasts increasingly express the complement factor C3, which can activate macrophages via their C3 receptors [13]. Furthermore, the finding that cardiac fibroblasts express MHC class II molecules for antigen presentation to CD4+ T cells upon interferon gamma stimulus and contribute to myocardial fibrosis, further strengthens the suggested fibroblast–immune cell interaction [158]. Together, these processes create a self‐perpetuating cycle in which aging exacerbates immune cell–fibroblast interactions, amplifying inflammation and fibrosis [159].

Adaptive immunity, specifically T‐cells, may further aggravate cardiac inflammaging. Adoptive transfer of aged, senescent T cells to immunodeficient RAG1 KO mice accelerates Ang II‐induced cardiovascular and renal fibrosis compared with young T‐cell transfer [160]. Likewise, adoptive cell transfer of T cells from aged animals [146] or transplantation of terminally differentiated CD4+ T cells in young mice promotes myocardial inflammation and leads to impaired cardiac function. Transplantation of these T cell populations in healthy mice caused rather mild effects on cardiac function [146] suggesting that spontaneous, heart‐directed immune responses during aging may arise, but that additional innate immune and intrinsic cardiac changes may contribute to the decline of age‐related myocardial inflammation and functional decline.

### 3.3 Inflammaging and Atherosclerosis

Particularly in vascular disease such as atherosclerosis, inflammation is well documented to causally contribute to disease progression. Indeed, anti‐inflammatory therapies targeting IL‐1β have been shown to attenuate disease progression, as demonstrated in the CANTOS trial [161]. Inflammation in atherosclerosis is generally thought to be initiated by cholesterol accumulation, oxidized lipids, reduced nitric oxide bioavailability, and increased reactive oxygen species. However, the extent to which these pro‐inflammatory signals are directly driven by aging remains incompletely understood. Although aging is a major risk factor for atherosclerosis, relatively few studies have specifically assessed its specific impact. Notably, aged hyperlipidemic mice exhibit a higher number of macrophages infiltrating the aortic wall compared with young animals [162]. In addition, aged mice on a high‐fat diet display increased arterial levels of IL‐6 and macrophage‐attracting chemokines compared with young counterparts [162]. Pro‐inflammatory, senescence‐associated CD4 + CD28‐CD57+ T‐cells were associated with higher load‐dependent arterial stiffness. Multiple pro‐inflammatory CD4+ and CD8+ T‐cell subpopulations were associated with both higher structural and load‐dependent arterial stiffness [163].

### 3.4 Role of Somatic Mutations in Inflammaging

Age is associated with an increased accumulation of somatic mutations and chromosomal aberrations in humans. Such mutations can drive the clonal expansion of hematopoietic cells, a process termed clonal hematopoiesis (CH). While initially regarded primarily as a precursor to leukemia, CH has been shown to confer only a modest risk for hematologic malignancies but is strongly associated with a higher incidence and progression of cardiovascular disease [164, 165, 166]. Age‐associated clonal expansion is frequently caused by mutations in encoding for epigenetic regulators such as DNMT3A and TET2, as well as less common mutations in ASXL1, TP53, and others. Most of these alterations promote inflammatory responses in macrophages and other immune cells, leading to immune dysfunction and thereby contributing to cardiovascular disease, including atherosclerosis and heart failure, as summarized in recent review articles [167, 168]. Although the precise mechanisms differ between mutations, single‐cell sequencing studies have revealed both shared and mutation‐specific gene expression changes in circulating blood cells of CH carriers [169]. TET2 mutations in particular enhance expression and release of pro‐inflammatory cytokines such as IL‐1β, activating the NLRP3 inflammasome thereby driving atherosclerosis [170, 171, 172] and NLRP3‐inhibition rescues adverse remodeling and cardiac hypertrophy [173]. In contrast, *DNMT3A* mutations appear less pro‐inflammatory and less strongly linked to atherosclerosis, but instead promote paracrine pro‐fibrotic signaling and aggravate cardiac fibrosis [174]. Notably, the senomorphic drug metformin reduces the clonal expansion of *DNMT3A* mutant hematopoietic stem cells, although it is unclear, if this is due to senescence‐associated mechanisms [175].

The most common age‐associated somatic alteration is mosaic loss of the Y chromosome (LoY) in leukocytes, which occurs in ~40% of men at the age of 70 [176]. LoY worsens the prognosis of several cardiovascular diseases, including atherosclerosis [177], aortic valve stenosis [178, 179], and heart failure [180, 181], as reviewed recently [182]. Mechanistically, LOY has been linked to enhanced fibrotic activation [180] and impaired clearance of senescent cells [183], thereby exacerbating cardiac and systemic aging. While these mechanisms may serve as therapeutic targets, to date no specific treatments are available.

### 3.5 Therapeutic Interventions Targeting Inflammaging

Inflammaging can be targeted through a range of strategies, encompassing both pharmacological interventions and well‐established lifestyle measures such as diet and physical activity (reviewed in [184]). This review will primarily highlight emerging mechanistic approaches, many of which are still at the experimental or preclinical stage, while only briefly summarizing the recognized benefits of lifestyle‐based interventions.

#### 3.5.1 Pharmacological Interventions

##### 3.5.1.1 Targeting Inflammaging

The first line of intervention against inflammation often involves the use of anti‐inflammatory drugs such as NSAIDs. However, their long‐term use is limited by significant risks and side effects. Safer alternatives include widely used medications such as metformin and statins, which not only exert anti‐inflammatory effects but also provide additional metabolic and cardiovascular benefits [185]. Despite these advantages, there remains a pressing need for more specific therapeutic strategies that can selectively target the underlying inflammatory pathways without compromising safety during chronic use. The first clinical trial to show beneficial effects of targeted anti‐inflammatory therapies in cardiovascular disease was the CANTOS trial, in which IL‐1β blockade resulted in a 26% relative risk reduction for the composite of nonfatal myocardial infarction, nonfatal stroke or cardiovascular death in patients with previous myocardial infarction [161]. Interestingly, this effect was most pronounced in patients with *TET2* clonal hematopoiesis [172]. Later trials showed benefits of colchicine treatment, an immunomodulatory drug primarily exerting anti‐inflammatory effects through NLRP3 inhibition, in chronic coronary syndromes and post‐AMI [186, 187]. However, it remains uncertain, if these effects are driven by inhibition of aging phenotypes or rather primary pro‐inflammatory processes involved in atherosclerosis and myocardial infarction. Subsequent work has to investigate if anti‐inflammatory treatment shows larger effects in aged populations. Of note, broad inhibition of inflammatory activation with non‐specific anti‐inflammatory drugs may inadvertently accelerate immunosenescence, that is, by impairing the clearance of senescent cells, and may also weaken immune responses to infections or vaccinations in the elderly. In addition, chronic anti‐inflammatory therapy could compromise immune surveillance and potentially increase cancer risk—although this was not observed in the CANTOS trial [188].

An alternative approach is to reprogram macrophages, restoring their function towards a reparative phenotype. This strategy not only limits excessive inflammation but also enhances the clearance of senescent cells. Such interventions have been investigated in cardiovascular disease models and may also help counteract inflammaging, thereby preventing disease progression. Examples include targeting RUNX1 in macrophages to promote cardiac recovery [189] or enhancing efferocytosis by upregulating “eat‐me” signals such as MERTK. For instance, augmentation of MerTK^+^ cardiac macrophages with LXR agonists reduced LPS‐mediated electrical instability and arrhythmias [87]. Moreover, Resolvin D1 has been shown to promote efferocytosis in aging by preventing senescent cell‐induced MerTK cleavage in a lung ischemia/reperfusion model [190].

In addition, targeting “don't‐eat‐me” signals such as SIRPα or CD47 has shown therapeutic potential. Systemic blockade of CD47 with antibodies against its receptor SIRPα reduced plaque vulnerability and lesion size in atherosclerosis models [191]. In a phase I trial, a humanized anti‐CD47 antibody decreased carotid artery inflammation, although anemia emerged as a side effect due to erythrophagocytosis of aged red blood cells [191]. To address this limitation, nanotherapies delivering a SHP‐1 inhibitor downstream of the CD47–SIRPα axis were developed and demonstrated the ability to reduce vascular inflammation without inducing anemia in large animal models [192]. Moreover, direct blockade of SIRPα itself has also been shown to reduce atherosclerosis [193].

Beyond the CD47–SIRPα pathway, the inhibitory receptor CD300f has been implicated in protecting against systemic aging. CD300f dampens myeloid cell activation, thereby limiting excessive inflammation through downregulation of cytokine release and phagocytic activity, while at the same time facilitating efferocytosis in a controlled, non‐inflammatory manner. By regulating inflammaging, metabolism, and cognitive decline, CD300f contributes to healthy aging, whereas knockout models display premature aging phenotypes [194]. Its function in the cardiovascular system remains to be established.

Clearance of senescent cells may also be impaired by posttranscriptional modifications, for example, glycosylation, which interferes with macrophage‐ or NK cell–mediated elimination. Notably, many senescent cell types upregulate the ST8 alpha‐N‐acetyl‐neuraminide alpha‐2,8‐sialyltransferase (*ST8SIA1*) gene, which contributes to the synthesis of disialylated ganglioside GD3. High surface expression of GD3 enables senescent cells to evade NK cell–mediated killing, thereby escaping immunosurveillance. Consistent with this, anti‐GD3 antibody therapy in mice prevented the development of bleomycin‐induced lung fibrosis and attenuated several age‐related disorders, including lung and liver fibrosis as well as osteoporosis [195].

One may additionally consider targeting adaptive immunity. Such strategies include immunomodulation through immunopeptides and autoantibodies in aging [196], which might also be relevant in light of recent reports on autoimmunity and T cell populations in heart failure. Another promising approach is the selective targeting of T cell subsets. For example, new immunosenescence‐directed strategies for preventing senescence‐associated pathological cardiac hypertrophy involve interfering with IL‐17 signaling. Both RAR‐related orphan receptor gamma (RORγt) inhibitors and IL‐17A neutralizing antibodies have been shown to prevent cardiac aging and dysfunction [197].

### 3.6 Repurposing Anti‐Diabetic Drugs

In recent years, the use of anti‐diabetic drugs in cardiovascular disease has emerged as a transformative area of research, revealing pleiotropic mechanisms that extend far beyond glycemic control and open new perspectives for targeting aging‐related processes. Sodium/glucose cotransporter 2 (SGLT2) inhibitors have been shown to exert beneficial effects on cellular senescence and inflammaging through multiple mechanisms beyond their primary role in glucose regulation [198]. These agents reduce markers of senescence such as p53, p16, and senescence‐associated β‐galactosidase, suppress the pro‐inflammatory SASP, and alleviate pathological aging in mice [199]. SGLT2 inhibitors activate AMPK and sirtuin 1 pathways, reduce mTOR and insulin/insulin‐like growth factor (IGF) signaling, mimic the effects of calorie restriction, and promote autophagy and mitochondrial health, all processes linked to anti‐aging effects.

The GLP‐1/GIP receptor agonist Tirzepatide also exerts anti‐inflammatory properties by reducing systemic inflammation and oxidative stress associated with aging and metabolic disease, and it interferes with senescent cardiac cells [104]. However, direct evidence for reducing senescent cells is less well established for GLP‐1 or GLP‐1/GIP agonists compared with SGLT2 inhibitors.

Importantly, the metabolic benefits of both drug classes, including improved insulin sensitivity, weight loss, and cardiovascular protection, may indirectly reduce senescence and inflammaging by enhancing metabolic homeostasis and lowering cellular stress.

#### 3.6.1 Supplements and Life Style Interventions

Ample experimental data support the anti‐aging effects of dietary interventions, for example, omega‐3 fatty acids, polyphenols (e.g., resveratrol, curcumin) or other phytochemicals exhibiting antioxidant, anti‐inflammatory, and autophagy‐enhancing effects that target core pathways involved in cellular senescence and tissue degeneration. These phytochemicals regulate key molecular players such as sirtuins, AMPK, NF‐κB, and mTOR, offering promise in delaying age‐associated pathologies and promoting longevity. However, there is no documented benefit in clinical studies for the prevention of cardiovascular aging.

In addition, anti‐inflammatory diets (e.g., a Mediterranean diet) and lifestyle interventions such as regular moderate exercise, caloric restriction, or intermittent fasting have well‐documented beneficial effects and reduce inflammaging [105]. For example, the randomized controlled CALERIE Phase 2 trial demonstrated significant improvements in aging biomarkers and insulin resistance after 2 years of moderate calorie restriction compared with a usual diet [200]. However, most studies investigated these interventions in patients with metabolic syndrome or demonstrated diabetes‐preventive effects, making it difficult to distinguish metabolic from specific anti‐aging effects. Therefore, prospective studies specifically designed to evaluate the clinically relevant impact of such interventions on cardiovascular aging are still lacking.

## 4 Outlook

Aging remains the most important yet unmodifiable risk factor for cardiovascular disease. Consequently, targeting cardiovascular aging represents an attractive strategy to improve long‐term cardiovascular health. Therapeutic approaches are diverse, ranging from the elimination of senescent cells and modulation of their secretory phenotype to interference with inflammaging and metabolic dysregulation as outlined above. Several drugs already in clinical use for other indications, such as metformin and other anti‐diabetic agents, have demonstrated beneficial effects on age‐related phenotypes, suggesting that cardiovascular protection may be, at least in part, linked to their anti‐aging properties. Nevertheless, strategies aimed at directly targeting senescence or chronic inflammation are still needed.

Major challenges remain. Senescent cells also fulfill important physiological functions, including tissue repair and tumor suppression. Similarly, interventions that blunt immune‐driven inflammation must carefully balance the protective roles of the immune system against pathogens with the prevention of chronic, destructive inflammation. Thus, indiscriminate deletion of senescent cells or broad suppression of inflammatory pathways carries inherent risks.

These concerns highlight the importance of developing more selective, biomarker‐guided, and possibly intermittent therapeutic strategies. Advances in precision medicine, single‐cell profiling, and the identification of senescence‐specific markers will be critical to guide such approaches. Ultimately, the success of anti‐aging drug development will depend on striking a balance between efficacy in reducing pathological aging and preserving essential homeostatic functions.

## Funding

This work was supported by Deutsche Forschungsgemeinschaft (SFB1366, SFB1531), Deutsches Zentrum für Herz‐Kreislaufforschung, Partner Site Project and H2020 European Research Council, Neuroheart.

## Conflicts of Interest

The authors declare no conflicts of interest.

## Data Availability

The authors have nothing to report.
